# A new dipole index of the salinity anomalies of the tropical Indian Ocean

**DOI:** 10.1038/srep24260

**Published:** 2016-04-07

**Authors:** Junde Li, Chujin Liang, Youmin Tang, Changming Dong, Dake Chen, Xiaohui Liu, Weifang Jin

**Affiliations:** 1 State Key Lab of Satellite Ocean Environment Dynamics, Second Institute of Oceanography, State Oceanic Administration, Hangzhou, China; 2 College of Physical and Environmental Oceanography, Ocean University of China, Qingdao, China; 3 Environmental Science and Engineering, University of Northern British Columbia, Prince George, British Columbia, Canada; 4 Oceanic Modeling and Observation Laboratory, Nanjing University of Information Science and Technology, Nanjing, China; 5 Department of Atmospheric and Oceanic Sciences, University of California, Los Angeles, California, USA

## Abstract

With the increased interest in studying the sea surface salinity anomaly (SSSA) of the tropical Indian Ocean during the Indian Ocean Dipole (IOD), an index describing the dipole variability of the SSSA has been pursued recently. In this study, we first use a regional ocean model with a high spatial resolution to produce a high-quality salinity simulation during the period from 1982 to 2014, from which the SSSA dipole structure is identified for boreal autumn. On this basis, by further analysing the observed data, we define a dipole index of the SSSA between the central equatorial Indian Ocean (CEIO: 70°E-90°E, 5°S-5°N) and the region off the Sumatra-Java coast (SJC: 100°E-110°E, 13°S-3°S). Compared with previous SSSA dipole indices, this index has advantages in detecting the dipole signals and in characterizing their relationship to the sea surface temperature anomaly (SSTA) dipole variability. Finally, the mechanism of the SSSA dipole is investigated by dynamical diagnosis. It is found that anomalous zonal advection dominates the SSSA in the CEIO region, whereas the SSSA in the SJC region are mainly influenced by the anomalous surface freshwater flux. This SSSA dipole provides a positive feedback to the formation of the IOD events.

The Indian Ocean Dipole (IOD) is a prominent air-sea coupled climate mode of inter-annual variability in the tropical Indian Ocean^1^,^2^ characterized by an opposite sea surface temperature anomaly (SSTA) in the eastern and western Indian Ocean, with the sea surface wind anomalies over the central equatorial Indian Ocean. During positive IOD (pIOD) events, the sea surface temperature (SST) is lower than normal off the Sumatra–Java coast but warmer than normal in the western tropical Indian Ocean, and vice versa for a negative IOD (nIOD) phase^3^. Due to the strong SSTA gradient across the Indian Ocean basin, the IOD can significantly modulate the atmospheric circulation, inducing a series of climate anomalies in many areas of the world, including the surrounding area of the Indian Ocean, South America, East Africa, Southeast Australia, Europe and Northeast Asia^4^,^5^,^6^,^7^,^8^,^9^. Significant impacts of the IOD on natural resources, the environment and societies have also been documented, in a variety of areas including water resources, agriculture, forestry, ecosystems, human health and other climate-sensitive sectors^10^.

Originally, the IOD was found and formalized in the field of the SST^1^. This is likely because the SST is usually the first target in exploring large-scale oceanic and atmospheric variability due to its importance to the upper ocean circulation, atmospheric circulation and weather and climate anomalies^11^,^12^,^13^. In addition, the inter-annual variability in the tropics often has the strongest signal in the SST, which also has the best observation record in oceanic data, providing a good basis to detect its large-scale anomaly structure^14^,^15^. However, in recent years, the importance of sea surface salinity (SSS) anomalies in linking the global hydrological cycle and climate anomalies has been well understood and addressed^16^. One principal explanation is that SSS controls the barrier layer variation^17^, defined as the difference between the isothermal layer depth (ILD) and the mixed layer depth (MLD), thereby further impacting the upper ocean circulation and thermodynamic structure.

Over the last decade, a number of studies have been conducted to investigate SSS anomalies (SSSA) associated with the IOD, using observations, models and reanalysis products. It was recognized that the SSSA often has significant out-of-phase variations between the positive and negative IOD events at some regions, such as the central equatorial Indian Ocean and off Sumatra-Java coast^18^,^19^,^20^,^21^,^22^,^23^,^24^,^25^. There have been considerable efforts to define an index to describe the dipole variability of the SSSA like the Dipole Mode Index (DMI) used to describe the variability for the SSTA. For example, a salt storage dipole index is defined using the SSS difference between (5°S-5°N,55°E-75°E) and (10°S-0°N,85°E-95°E)^26^. A Zonal Salinity Index (ZSI) is defined using another two boxes of (5°S-5°N,80°E-90°E) and (10°S-0°N,90°E-105°E)^21^. Recently, an index is defined using the averaged SSSA over the region off Sumatra (10°S-0°N,95°E-105°E) to capture the out-of-phase variability of the salinity there at different IOD phases; this index is called the Dipole Mode Index of Salinity (DMIS)^22^. However, none of these indices can well characterize the spatial dipole structure of the SSSA across the tropical Indian Ocean; there are several likely reasons for this limitation. First, the existing salinity data, observations or reanalysis products, which were used to define the indices in literature, are sparse in space and short in time, making it difficult to precisely detect the locations of the dipole centres of the SSSA variability, and a dipole index is highly dependent on the chosen location of the dipole centres. Second, these indices were mostly motivated by examining the impact of the IOD on the SSSA and were derived from the analysis of the SSTA-defined IOD phases, rather than by emphasizing the SSSA variability itself. This assumes the SSSA to be an intrinsic slaver of the SSTA. In the following analysis, we will find that the SSSA variability is impacted not only by the SSTA but also by other forcing that probably drives them both simultaneously. Another concern regarding such an IOD phase-based index is its ability to characterize the variability at the negative dipole phase due to the asymmetry of the IOD. Third, these indices used data from all seasons, and the seasonality of the SSSA dipole was not well considered. It was found that the IOD variance has a significant seasonal variation with the maximum during the autumn.

In this study, a high-resolution eddy-resolving regional ocean model (ROMS), which produces a 33-year high-quality simulation of the SST and SSS anomalies from 1982 to 2014 (Figs S1 and S2), is well configured for the tropical Indian Ocean. An IOD-relevant dipole pattern of the SSSA is first detected and formalized using the modelled SSS product in autumn and is further confirmed by the ARGO and SODA datasets. The possible physical mechanism responsible for the SSS dipole structure is also investigated by diagnosing the mixed-layer model of salinity variation in this study.

## Results

### Sea surface salinity dipole

**Figure 1:**
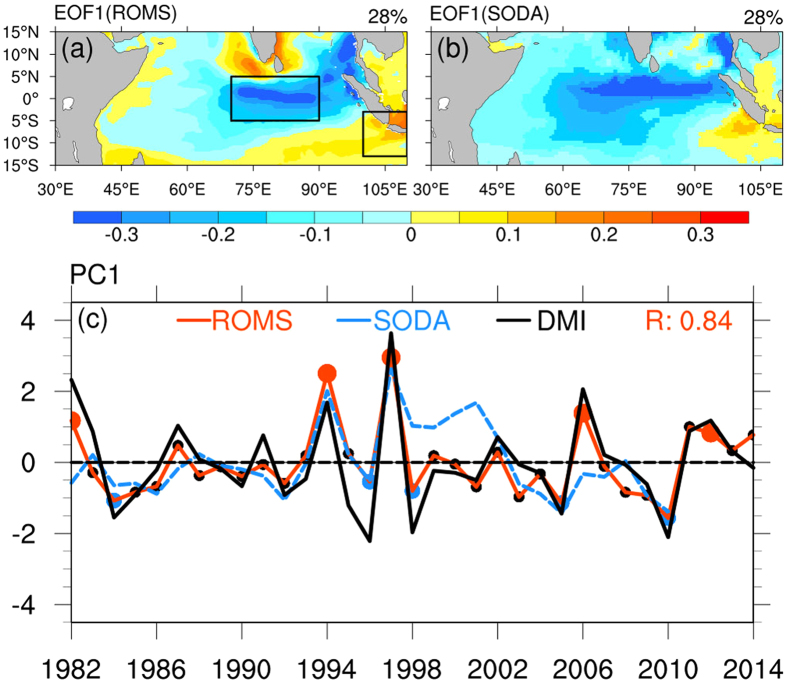
Spatial pattern of the first EOF mode and its corresponding principal component time series for SSS anomalies obtained using boreal autumn data (September-November, SON) from 1982 to 2014. (**a**,**b**) are the first EOF patterns from ROMS and SODA, respectively. The black boxes in (**a**) show the dipole locations in the central equatorial Indian Ocean (CEIO: 70°E–90°E, 5°S–5°N) and the Sumatra-Java coast (SJC: 100°E–110°E, 13°S–3°S). The maps were generated in The NCAR Command Language (Version 6.3.0) [Software]. (2016). Boulder, Colorado: UCAR/NCAR/CISL/TDD. http://dx.doi.org/10.5065/D6WD3XH5. (**c**) Time series of the first EOF principal component (PC1) from ROMS (solid red line) and SODA (dashed blue line), and the corresponding SON mean DMI from ROMS (solid black line). Five positive IOD events (1982, 1994, 1997, 2006 and 2012) and five negative IOD events (1984, 1996, 1998, 2005 and 2010) are indicated on ROMS PC1 with red dots and blue dots, respectively, and black dots for normal years. The correlation coefficient between ROMS PC1 and ROMS DMI is indicated.

An EOF analysis is conducted on the ROMS SSS simulation. Considering the seasonality of the IOD, we only use SON (Sep-Oct-Nov) data from 1982 to 2014 in the EOF analysis. Figure 1a shows the first EOF mode, accounting for 28% of the total variance, which clearly displays an opposite anomaly between the central equatorial Indian Ocean (CEIO: 70°E–90°E, 5°S–5°N) and the region off the Sumatra–Java coast (SJC: 100°E–110°E, 13°S–3°S). The variation of SON SSSA between CEIO and SJC is out-of-phase, with a statistically significant correlation of −0.61 at the 95% confidence level. Such an opposite variation in the SSSA between the two regions, with the negative value at the CEIO and the positive value at the SJC, can be defined as a positive phase of a dipole pattern, and vice versa for a negative phase, as in the IOD in the SST anomaly. Fiure. 1b shows the first EOF mode from the SODA SSS reanalysis dataset, also accounting for 28% of the total variance. A similar dipole pattern to Fig. 1a is also found, confirming that the dipole pattern of SSSA is not data dependent. We also repeated the EOF analysis using ECMWF ORAS4 dataset, and obtained similar results (not shown).

To explore the seasonality of SSSA variability, similar EOF analyses are performed for other seasons. It is found that the dipole pattern of SSSA is the most significant in boreal autumn, followed by the winter, and the dipole pattern is absent in the spring and summer (Fig. S3). To our best knowledge, this is the first work to extract a dipole mode using seasonal SSSA data, which is different from previous works using all-seasons SSS data. We also used the SSS of all months during the 33-year period and found the dipole is not as striking as in Fig. 1a,b.

To examine the relationship between the SSSA dipole mode and the SSTA IOD, we compare the time series of the first EOF mode, i.e., the first principal component (PC1), against the model DMI, as shown in Fig. 1c. A good relationship is observed in this figure with a high correlation coefficient of 0.84 between the model SSSA PC1 and the model SSTA DMI. If the IOD event is defined by a DMI greater than 1.0 standard deviation (std) for at least 5 months, there are five positive IOD events (1982, 1994, 1997, 2006 and 2012) and five negative IOD events (1984, 1996, 1998, 2005 and 2010) during the 33-year period. It can be observed in Fig. 1c that the SSSA PC1 usually positively peaks at pIOD events, for example, at 1997 when both exceeded 3.0 standard deviations; whereas the SSSA PC1 also shows large negative anomalies at nIOD events, indicating that the SSSA dipole pattern almost co-occurs with the IOD event.

**Figure 2:**
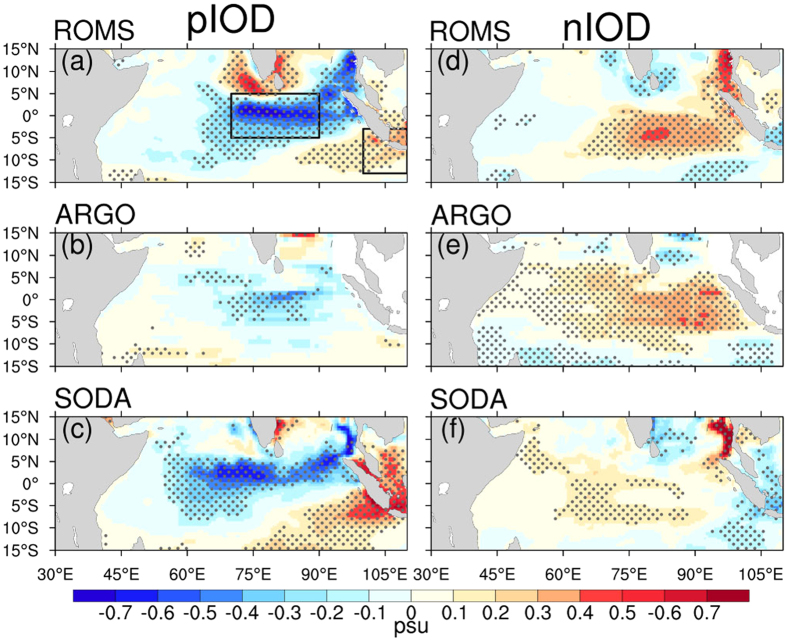
Composited SON mean SSS anomalies during IOD events. (**a**–**c**) comprise positive IOD events from ROMS (1982, 1994, 1997, 2006 and 2012), ARGO (2006 and 2012) and SODA (1982, 1994, 1997 and 2006), respectively. (**d**–**f**) Same as (**a**–**c**), but for negative IOD events from ROMS (1984, 1996, 1998, 2005 and 2010), ARGO (2005 and 2010) and SODA (1984, 1996, 1998, 2005 and 2010). The black boxes in (**a**) are the same as Fig. 1a. Gray stipples in (**a**–**f**) indicate anomalies that are statistically significant at the 95% confidence level. The maps were generated in The NCAR Command Language (Version 6.3.0) [Software]. (2016). Boulder, Colorado: UCAR/NCAR/CISL/TDD. http://dx.doi.org/10.5065/D6WD3XH5.

The strong dipole signal of the SSSA and its relationship with the IOD can be further revealed by composite analysis, which is often used to explore the IOD-relevant features^7^,^14^,^15^. Figure 2 shows the autumn SSSA composite maps for the positive and negative IODs from the ROMS model simulation (5 positive and 5 negative IODs as mentioned above), the ARGO data (2 positive events of 2006 and 2012 and 2 negative events of 2005 and 2010) and the SODA (4 positive events: 1982, 1994, 1997 and 2006 and 5 negative events: 1984, 1996, 1998, 2005 and 2010). Note a dipole pattern similar to Fig. 1a,b is visible in Fig. 2, with negative SSS anomalies at CEIO and positive anomalies at SJC during the positive IOD events and vice versa. This is particularly apparent for the ROMS and SODA data. The weak intensity of the dipole pattern in the ARGO data is likely due to sparse data samples and few IOD events used in the composite analysis, especially because the latter excludes several of the strongest IOD events (e.g., 1982, 1994 and 1997 for pIOD and 1996 and 1998 for nIOD).

A striking feature in Fig. 2 is the asymmetric structure of the SSSA dipole mode; the dipole signal is much stronger in the pIOD than in the nIOD. Such an asymmetry of the SSSA dipole mode is also noticed in previous work^23^. This is likely the main reason why the SSSA dipole indices that were defined based on the IOD composite analysis in previous works are unable to well characterize their negative phases^21^,^22^.

### Sea surface salinity anomalies index

As discussed above, a SSSA dipole structure has been found in the first EOF mode and the composite map, which co-occurs with the IOD. Naturally, an index is expected to well characterize the variability of the SSSA dipole mode. From the point of view of a coupling system, the SSTA and SSSA may occur in an interaction mode, with mutual impact and similar external forcing. Thus, in defining this SSSA index (SSSAI), we consider not only the signal strength of the dipole variability but also the link to the SSTA IOD index. The importance of the latter was not well reflected in previous SSSA dipole indices as indicated by weak correlation coefficients of the SSSA indices related to the IOD index, and poor representation of the SSSA for negative IOD events^21^,^22^,^26^. We choose different pole boxes to define the dipole index and to examine their variances and their correlations to DMI. After many trials and these sensitivity analyses, we define the index by the difference between the averaged SSSA over the 70°E–90°E, 5°S–5°N of the central equatorial Indian Ocean and that over the 100°E–110°E, 13°S–3°S of the region off the Sumatra–Java coast.

**Figure 3:**
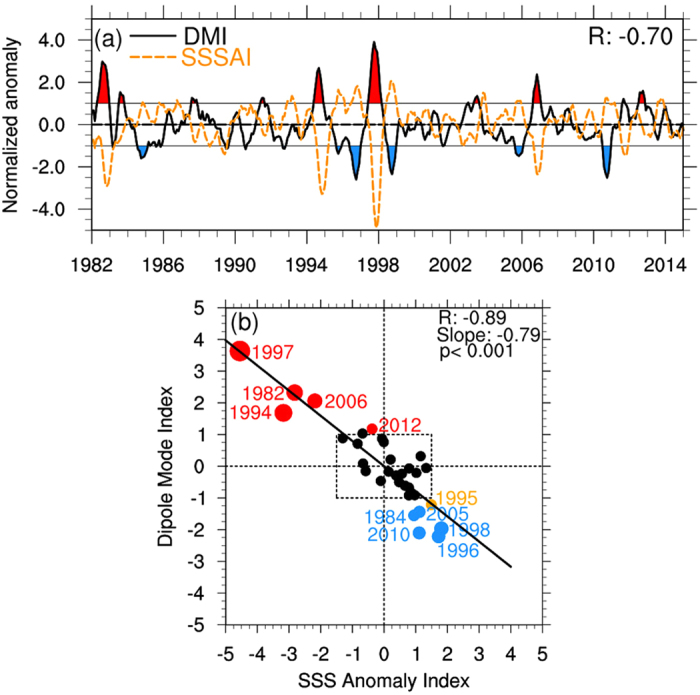
Relationship between the Dipole Mode Index (DMI) and SSS anomalies index (SSSAI). (**a**) The DMI (black solid line) and SSSAI (orange dashed line) from 1982 to 2014. Both indices are normalized by their respective standard deviations (std); those values greater than 1.0 std of DMI are highlighted in red, while those less than 1.0 std are highlighted in blue. The two indices are also filtered by a bandpass filter of 5–84 months. (**b**) Scatter diagram of SON mean DMI versus SSSAI during 1982–2014. The black dashed box indicates the departure from the mean of ±1.5 std for an SSS anomaly and ±1.0 std for an SST anomaly. The linear regression is conducted with the slope, correlation and p value indicated. Years defined as pIOD and nIOD events are highlighted with red and blue dots, respectively, and the dot size represents the intensity of the IOD event. Normal years are indicated with black dots, except for an orange dot for 1995. All plots were generated in The NCAR Command Language (Version 6.3.0) [Software]. (2016). Boulder, Colorado: UCAR/NCAR/CISL/TDD. http://dx.doi.org/10.5065/D6WD3XH5.

The variation of the SSSAI and the DMI for the period from 1982 to 2014 is shown in Fig. 3a, both from the ROMS simulation, filtered by a bandpass filter of 5–84 months and normalized by their individual standard deviations, as performed in other works^1^. Apparently, the variation of the SSSAI is out of the phase with the DMI, and both have a strong negative correlation of −0.70, which indicates the co-existence of the two dipole patterns. Similar results can also be obtained in the SODA data; for example, the correlation coefficient between the SSSAI and DMI is −0.64. Such a good relationship between the SSSAI and the DMI is also very strong in a composite analysis, as shown in Fig. S4, where the composites of the DMI and SSSAI are obtained for the period from the previous year to the following year of 5 positive and 5 negative IOD events. The good relationship between the SSSAI and the DMI can be further enhanced in the autumn when the IOD is strongest. Figure 3b is a scatter plot of the SON mean DMI and SSSAI for the period from 1982 to 2014. As expected, the robust relationship, up to a correlation coefficient of −0.89, is noteworthy.

The black dashed box in Fig. 3b is drawn to surround the departures from the mean of ±1.5 std for the SSS anomaly and ±1.0 std for the SST anomaly, which cover almost all the non-IOD years (black dots) during this period, except 1995 (orange dot). Five pIOD years and five nIOD years, all outside the box, are drawn in red dots and blue dots, respectively. In this scatter plot, several interesting features can be observed. First, the stronger IOD events have larger SSSAI values and vice versa. Second, the SSSA amplitude in the positive and negative IOD events are asymmetric, with most pIOD events having a larger amplitude (>2.0 std) compared with the nIOD events (<2.0 std). Third, a linear regression of the DMI against the SSSAI, as shown in the black solid line, has a slope of −0.79, indicating that the change of SSSA with 1.0 std (0.18 psu) is equivalent to a change of SSTA with −0.79 std (0.5 °C). The co-existence and co-occurrence of SSSA and SSTA may provide a new perspective to define the IOD event, which will be taken under study.

A further examination of Fig. 3a reveals that the variation of SSTA leads that of the SSSA by 1 month. This is especially apparent during the positive IOD events (Fig. S4a). There are likely two reasons for this time lag of SSSA; one is the impact of the SSTA on SSSA and the other is that the SSTA responds earlier to the wind anomalies than does the SSSA. In the following discussion, we will explore the mechanism for both possible reasons, in particular the former one.

### Dynamical mechanisms governing SSS dipole mode

To examine the physical processes responsible for the genesis of the SSSA dipole mode, we consider a mixed layer salt budget model (see Methods), from which the variation of salinity anomalies in the mixed layer is found to be contributed from the four following processes: zonal horizontal advection (ZADV), meridional horizontal advection (MADV), net surface freshwater flux (SFRE) and vertical physics in the form of entrainment (ENTR). Each process on the right hand side of equation (1) includes three terms. In the following discussion, each term of the first three processes is diagnosed, but ENTR is treated as an integral due to the relatively small contributions of each of its individual items. Considering the developing time of the variation that results in salinity anomalies in the autumn, we diagnosis the above processes of August-September of the IOD events. In a previous study, it was found that the phase of salinity anomalies delays the salinity variation of the mixed layer by 1–2 months^23^.

**Figure 4:**
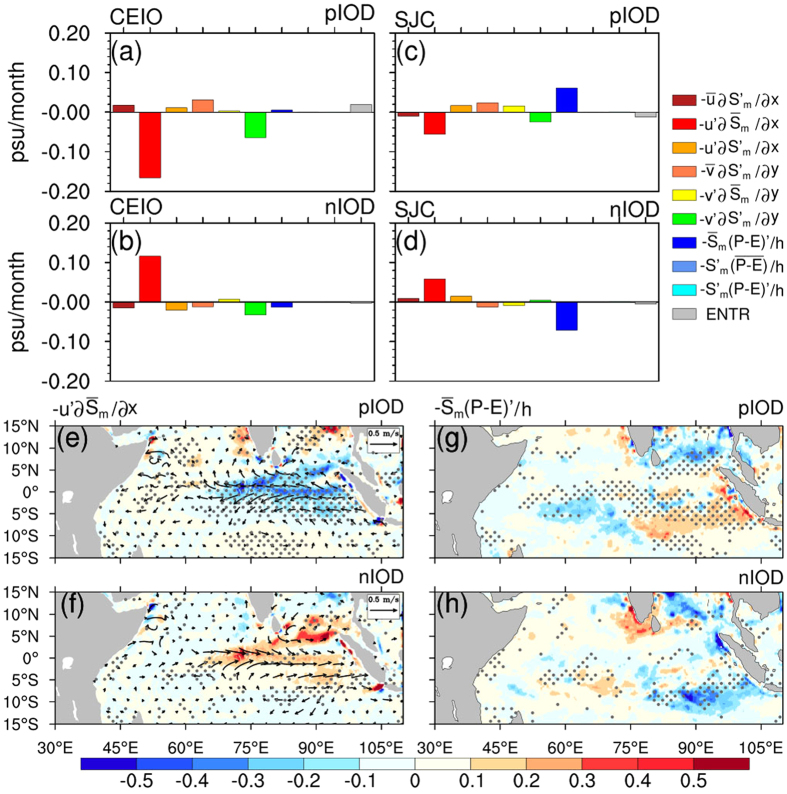
Composited salinity budget components averaged over August–September during IOD events. (**a**,**b**) are salinity budget components averaged within the CEIO region for pIOD and nIOD events, respectively. (**c**,**d**) Same as (**a**,**b**), but for SJC. (**e**,**f**) are the spatial distribution of 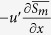 and surface current anomalies for composited pIOD and nIOD events, respectively. (**g**,**h**) Same as (**e**,**f**), but for 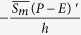. Gray stipples in (**e**–**h**) indicate anomalies that are statistically significant at the 95% confidence level. The maps were generated in The NCAR Command Language (Version 6.3.0) [Software]. (2016). Boulder, Colorado: UCAR/NCAR/CISL/TDD. http://dx.doi.org/10.5065/D6WD3XH5.

Figure 4a shows the composite of the salinity budget components of August - September averaged over the five pIOD events. As demonstrated in a previous study^23^, it is clear that the anomalous zonal advection of the mean mixed layer salinity, 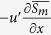, dominates the growth of the negative salinity anomalies in the CEIO region, and the anomalous meridional advection of the anomalous mixed layer salinity, 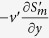, also makes a secondary contribution. Both 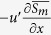 and 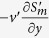 decrease the SSS in the CEIO region, which can also be observed for each pIOD event (Fig. S5a). However, during the nIOD period, the contribution of 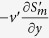 offsets the SSS increases by 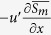, which itself is smaller in amplitude than that during the pIOD events (Fig. 4b, Fig. S6a). This may account for the asymmetric SSSA in the CEIO region during the positive and negative IOD events. For the SJC region, the dominant component is completely different. As shown in Fig. 4c and Fig. S5b, the anomalous surface freshwater flux of the mean mixed layer salinity, 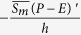, dominates the growth of the positive salinity anomalies during pIOD events. However, 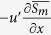 plays a comparable role in the opposite direction and dampens the growth. As expected, an opposite situation occurs during nIOD events, namely that, 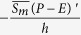 tends to freshen the SJC region, while 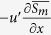 tends to impede it (Fig. 4d, Fig. S6b). The opposite contributions of the two comparable components in the SJC region leads to small SSSA variability there, thus the SSSAI is mainly controlled by the SSSA over the CEIO region.

In short, the SSSA dipole is governed by two dominant terms, the anomalous zonal advection of the mean mixed layer salinity 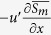 and the anomalous surface freshwater flux of the mean mixed layer salinity 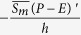. Figure 4e–h show the composite of the two items and the surface current anomalies, averaged over the five pIOD and five nIOD events. During the pIOD events, the SST off the Sumatra-Java coast became cooler than normal. Consequently, an anomalous west-east SST gradient enhances the easterly winds over the central and eastern equatorial Indian Ocean and off Sumatra-Java coast. This drives anomalous zonal currents that advect fresh water from the Sumatra-Java coast and the Bay of Bengal into the central and eastern equatorial Indian Ocean^21^ (Fig. 4e). Because of the asymmetric distribution of salinity in the western (high) and eastern (low) equatorial Indian Ocean, the climatological zonal mixed layer salinity gradient, 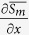, is negative in the equatorial Indian Ocean all year^23^. Therefore, the anomalous zonal current leads to negative 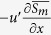 anomalies in the CEIO region during pIOD events (Fig. 4e). Moreover, a surface cooling suppresses rainfall off the Sumatra-Java coast, which contributes the most to the positive 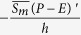 anomalies in the SJC region (Fig. 4g). For nIOD events, similar processes operate, however, in an opposite direction. Stronger than normal westerlies reinforce the Wyrtki Jet in the equatorial Indian Ocean and bring high-salinity water from the Arabian Sea to the central and eastern equatorial Indian Ocean. This in turn leads to the positive 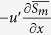 anomalies in the CEIO region (Fig. 4f). In addition, the warmer SST anomalies increase precipitation off Sumatra-Java coast, resulting in negative 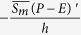 anomalies in the SJC region (Fig. 4h).

## Discussion

The inter**-**annual variability of the SSSA in the tropical Indian Ocean has received wide attention in recent years due to its profound influence on oceanic circulation and its inherent association with the IOD SSTA. There has been interest and efforts to define a salinity index to characterize a dipole structure of the SSSA like the DMI in the SSTA. In this study, we first apply EOF analysis to a high-quality model simulation and then to the observations, which identify the centre of the SSSA dipole using the autumn data. On this basis, an SSSA index is defined using two centres, one in the CEIO and one in the SJC region. Compared with previous SSSA indices, our SSSAI has several advantages, as outlined in Table 1. First, this index addresses the SSSA variability itself, from which the seasonality is considered in the dipole analysis, and the locations of dipole centres are deliberately detected using EOF analysis. Thus, it captures more striking dipole variability and has a larger standard deviation than others. Second, it can well characterize the SSSA dipole variability at its negative phase and can precisely describe the asymmetry of the SSSA during positive and negative IOD events, as indicated by the large slope amplitudes at both phases and a ratio of the positive to negative phase greater than 1.0 (negative slope value means a negative relationship between the SSSA index and the DMI). Third, the link between the SSSA and SSTA is carefully considered in defining this index so that it can have a much higher correlation with the DMI, especially during boreal autumn, thus building an intrinsic coherence between the SSSA and SSTA during the IOD events. The defined SSSAI can be used as a proxy equivalent to DMI to represent IOD events. Thus, in terms of the representation of the SSSA dipole variability itself and the intrinsic co-existence with the DMI, the index defined in this work should be the best to date.

Further, the evidence from the model simulation and observations are presented to support a hypothesis mechanism of co-occurrence between the SSSA and SSTA dipole by diagnosing the salt budget equation of the mixing layer. It is found that the anomalous zonal advection of the mean mixed layer salinity dominates the SSS anomalies in the CEIO region, whereas the anomalous surface freshwater flux of the mean mixed layer salinity plays an important role in the SSS variabilities in the SJC region.

An interesting question is why there is a co-existence of the SSSA dipole and SSTA dipole. Is their co-existence due to one quick impacting on the other or due to the control under common physical processes during IOD events? A comparison shows a short delay of the SSSAI to the DMI by approximately 1–2 months, suggesting that while the SSSA dipole mode is most probably excited by the physical processes that dominate the dipole pattern of the SSTA, the SSTA can impact the SSSA. As argued above, during pIOD events, cold waters off the Sumatra-Java coast can enhance easterly winds, driving anomalous zonal currents in the central and eastern equatorial Indian Ocean. This leads to low SSS anomalies in the CEIO region. Meanwhile, reduced rainfall increases SSS in the SJC region.

On the other hand, SSSA can also have an impact on the SSTA. For example, the salinity gradients significantly influence horizontal currents through their contribution to pressure gradients^27^, thus the anomalous SSS gradient between the CEIO and SJC regions in turn reinforces the westward-flowing currents and coastal upwelling, further cooling the SST off the Sumatra-Java coast. The opposite arguments can be applied to the impact of the negative phase of the SSSA dipole to nIOD events. Namely, the SSSA dipole provides a positive feedback to the formation of the SSTA dipole.

In short, the interaction and mutual influences of the SSSA and SSTA, under the control of the common factors during the IOD events, jointly contribute the co-existence of their dipole modes. A detailed and precise process of the co-existence of the two dipoles should be further studied, with a comprehensive understanding of the mechanism of the common factors that control the IOD, which is under study.

## Methods

### Definition

A DMI is defined by the difference of the averaged SSTA between the western (IODW: 50° E-70° E, 10° S-10° N) and the eastern (IODE: 90° E-110° E,10° S-0°) equatorial Indian Ocean. An SSSAI is defined by the difference of the averaged SSSA between a region in the central equatorial Indian Ocean (CEIO: 70°E–90°E,5°S–5°N) and a region off the Sumatra-Java coast (SJC: 100°E–110°E,13°S–3°S). An IOD event is defined by a DMI greater than 1.0 standard deviation for at least 5 months, from which five positive IOD events (1982, 1994, 1997, 2006 and 2012) and five negative IOD events (1984, 1996, 1998, 2005 and 2010) are selected during the period from 1982 to 2014 in this study (Fig. S2). The mixed layer depth is defined as the depth where the increase of the potential density, compared to that at 10 m depth, corresponds to a temperature decrease of 0.2 °C^28^.

### Data

ARGO monthly gridded SSS data from January 2004 to December 2014, with a resolution of 1° × 1°, provided by the Scripps Institution of Oceanography (http://www.argo.ucsd.edu/Gridded_fields.html) are used for validation purposes. The monthly optimum interpolation sea surface temperature (OISST) from January 1982 to December 2014 with a spatial resolution of 0.25°×0.25°, provided by NOAA (https://www.ncdc.noaa.gov/oisst), is used to construct the DMI. The Simple Ocean Data Assimilation 2.2.4 (SODA) monthly reanalysis product from December 1978 to December 2010 (http://sodaserver.tamu.edu/assim/SODA_2.2.4/) is used to provide the model’s initial and boundary condition, and the SODA SSS is also used for SSSAI validation and analysis. The Estimating the Circulation & Climate of the Ocean (ECCO) reanalysis product from January 2011 to December 2014 is also used to provide the model boundary condition (http://ecco.jpl.nasa.gov/). The 6-hourly surface atmospheric variables from the Climate Forecast System Reanalysis (CFSR) 93.0 over January 1979-March 2011 (http://rda.ucar.edu/datasets/ds093.0/) and from the Climate Forecast System Version 2 (CFSv2) (http://rda.ucar.edu/datasets/ds094.0/) over April 2011-December 2014 provided by NCEP are used to force the ocean model. The monthly climatological runoffs are from the Climate and Global Dynamics Laboratory (CGDL) (http://www.cgd.ucar.edu/cas/catalog/surface/dai-runoff/).

### Model

The Regional Ocean Modeling System (ROMS) developed by Rutgers University is used in this study. ROMS is a free-surface, terrain-following, and primitive equations ocean model, which has been widely used in the study of oceanic processes, simulations and predictions^29^,^30^. The domain in the model is configured within 30°E-110°E and 30°S-30°N. The horizontal resolution is 1/8° * 1/8°, and the model has 32 vertical levels with the vertical s-coordinate parameters *θ*_*s*_ = 3.0, *θ*_*b*_ = 0.3, which are the S-coordinate surface and bottom control parameter respectively, and the newly defined function of the vertical levels (Vtransform = 2, Vstretching = 4, which are used to select the vertical transform equation and stretching function respectively). The minimum depth at which a higher resolution in the upper layer of the ocean is defined with the parameter *h*_*c*_ = 100*m*, which is a positive thickness controlling the stretching, and the maximum depth is 5000 m. A 2 arc-minute global relief model of Earth’s surface that integrates land topography and the ocean bathymetry dataset (ETOPO2) is used to provide the topography, which is smoothed with the slope parameter 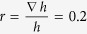, which is used for topography smoothing. The northern and western boundaries are bounded by land, whereas mixed boundary conditions are used along the southern and eastern open boundaries.

This model is initiated from the oceanic state in December of 1978 from SODA and then forced by CFSR and CFSv2. Near the boundaries, temperature, salinity, velocities and sea surface height (SSH) are relaxed to monthly SODA and ECCO. Considering the spin-up of the model, in this study, we discard the first three years and analyse the model simulation from January 1982 to December 2014. There is no relaxation of the SSS to the observed climatology in the experiment, but we add the continental freshwater forcing from CGDL to produce more realistic seasonal and inter**-**annual SSS variations.

The model has a good simulation for the tropical Indian Ocean SSTA and SSSA, as evidenced by Fig. 1 and Figs S1 and S2. For example, the DMI from the ROMS and the OISST show a high correlation of 0.86, exceeding the 95% significance level. This allows us to use the high-quality simulation to analyse the SSSA variability in this study.

### Mixed layer salinity budget analysis

The salinity budget equation for the mixed layer can be written as follows^31^:


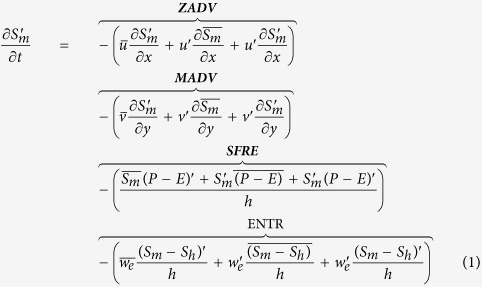


where S_m_ is the mixed layer salinity, u and v are the zonal and meridional ocean current velocities averaged over the mixed layer, P is precipitation, E is evaporation, S_h_ is the salinity at the base of the mixed layer, h is the mixed layer depth, and w_e_ (positive when upward) is the entrainment velocity, which is associated with the vertical velocity w_h_ at the base of the mixed layer and mixed-layer deepening^32^. The superscript prime and overbar denote anomalous and climatological mean seasonal cycle, respectively. The anomalous variables in Equation (1) are filtered by a bandpass filter of 5–84 months. Equation (1) expresses that the anomalous salinity tendency in the ML is balanced by the zonal horizontal advection (ZADV), meridional horizontal advection (MADV), net surface freshwater flux (SFRE) and vertical physics in the form of entrainment (ENTR)^23^.

### Statistical analysis

Linear regression and correlation analysis are used to quantify the strength of the relationship between the variables (e.g., the DMI and SSSAI), and a two-tailed Student’s t-test is used to test their significance. All statistical analyses in this study have significance at the 95% confidence level.

### Graphics software

All maps and plots were produced using: The NCAR Command Language (Version 6.3.0) [Software]. (2016). Boulder, Colorado: UCAR/NCAR/CISL/TDD. http://dx.doi.org/10.5065/D6WD3XH5.

## Additional Information

**How to cite this article**: Li, J. *et al*. A new dipole index of the salinity anomalies of the tropical Indian Ocean. *Sci. Rep.* **6**, 24260; doi: 10.1038/srep24260 (2016).

## Supplementary Material

Supplementary Information

## Figures and Tables

**Table 1:** The standard deviation, the correlation coefficient with the DMI, and the regression coefficient to DMI, of the SON-averaged SSSA Indices defined in literature and in this study.

Index	Standard deviation	Correlation coefficient	Regression coefficient (slope) (positive phase)	Regression coefficient (slope) (negative phase)
SHI	0.15	−0.01	−0.17	0.21
ZSI	0.20	−0.75	−0.96	−0.51
DMIS	0.10	−0.03	0.13	−0.22
SSSAI	0.26	−0.89	−1.15	−0.80

Table shows the standard deviation (1^st^ column), the correlation coefficient with the DMI (2^nd^ column), the regression coefficient to the DMI at positive IOD phase (3^rd^ column), and the regression coefficient at negative IOD phase (4^th^ column). SHI is the index defined by (5°S-5°N,55°E-75°E) and (10°S-0°N,85°E-95°E)^26^; ZSI is the index defined by (5°S-5°N,80°E-90°E) and (10°S-0°N,90°E-105°E)^21^; DMSI is the index defined by (10°S-0°N,95°E-105°E)^22^; SSSAI is the index defined by (5°S-5°N,70°E-90°E) and (13°S-3°S,100°E-110°E) in this work. All computations here used the model data from ROMS.

## References

[b1] Saji N., Goswami B. N., Vinayachandran P. & Yamagata T. A dipole mode in the tropical Indian Ocean. Nature 401, 360–363 (1999). 16862108 10.1038/43854

[b2] Webster P. J., Moore A. M., Loschnigg J. P. & Leben R. R. Coupled ocean–atmosphere dynamics in the Indian Ocean during 1997–98. Nature 401, 356–360 (1999). 16862107 10.1038/43848

[b3] Saji N. & Yamagata T. Structure of SST and surface wind variability during Indian Ocean Dipole mode events: COADS observations. J. Clim. 16, 2735–2751 (2003a).

[b4] Cai W., Cowan T. & Sullivan A. Recent unprecedented skewness towards positive Indian Ocean Dipole occurrences and its impact on Australian rai nfall. Geophys. Res. Lett. 36, L11705, doi: 10.1029/2009GL037604 (2009).

[b5] Saji N. & Yamagata T. Possible impacts of Indian Ocean Dipole mode events on global climate. Clim. Res. 25, 151–169 (2003b).

[b6] Cai W., Van Rensch P., Cowan T. & Hendon H. H. Teleconnection pathways of ENSO and the IOD and the mechanisms for impacts on Australian rainfall. J. Clim. 24, 3910–3923 (2011).

[b7] Qiu Y., Cai W., Guo X. & Ng B. The asymmetric influence of the positive and negative IOD events on China’s rainfall. Sci. Rep. 4, 4943, doi: 10.1038/srep04943 (2014). 24828947 PMC4021319

[b8] Ummenhofer C. C. . What causes southeast Australia’s worst droughts? Geophys. Res. Lett. 36, L04706, doi: 10.1029/2008GL036801 (2009).

[b9] Kripalani R., Oh J. & Chaudhari H. Delayed influence of the Indian Ocean Dipole mode on the East Asia–West Pacific monsoon: Possible mechanism. Int. J. Climatol. 30, 197–209 (2010).

[b10] Hashizume M., Chaves L. F. & Minakawa N. Indian Ocean Dipole drives malaria resurgence in East African highlands. Sci. Rep. 2, 269, doi: 10.1038/srep00269 (2012). 22355781 PMC3280600

[b11] Vecchi G. A. & Harrison D. In Earth’s Climate: The Ocean-Atmosphere Interaction, Vol. 147 (eds Wang C. Z., Xie S. P. & Carton J. A.), 247–259 (American Geophysical Union, 2004).

[b12] Clark C. O., Cole J. E. & Webster P. J. Indian Ocean SST and Indian summer rainfall: Predictive relationships and their decadal variability. J. Clim. 13, 2503–2519 (2000).

[b13] Gao L. . Rainfall asymmetry in the southeast Indian Ocean between positive and negative IODs and its local impact. Atmos. Sci. Lett. 15, 127–133 (2014).

[b14] Weller E. & Cai W. Meridional variability of atmospheric convection associated with the Indian Ocean Dipole Mode. Sci. Rep. 4, 3590, doi: 10.1038/srep03590 (2014). 24395079 PMC3882748

[b15] Ng B., Cai W., Walsh K. & Santoso A. Nonlinear processes reinforce extreme Indian Ocean Dipole events. Sci. Rep. 5, 11697, doi: 10.1038/srep11697 (2015). 26114441 PMC4481856

[b16] Lagerloef G. S. E. Introduction to the special section: The role of surface salinity on upper ocean dynamics, air-sea interaction and climate. J. Geophys. Res. 107, 8000, doi: 10.1029/2002JC001669 (2002).

[b17] Qiu Y., Cai W., Li L. & Guo X. Argo profiles variability of barrier layer in the tropical Indian Ocean and its relationship with the Indian Ocean Dipole. Geophys. Res. Lett. 39, L08605, doi: 10.1029/2012GL051441 (2012).

[b18] Vinayachandran P. N. & Nanjundiah R. S. Indian Ocean sea surface salinity variations in a coupled model. Clim. Dyn. 33, 245–263 (2009).

[b19] Du Y. & Zhang Y. Satellite and Argo observed surface salinity variations in the tropical Indian Ocean and their association with the Indian Ocean Dipole Mode. J. Clim. 28, 695–713 (2015).

[b20] Sharma R., Agarwal N., Basu S. & Agarwal V. K. Impact of satellite-derived forcings on numerical ocean model simulations and study of sea surface salinity variations in the Indian Ocean. J. Clim. 20, 871–890 (2007).

[b21] Thompson B., Gnanaseelan C. & Salvekar P. Variability in the Indian Ocean circulation and salinity and its impact on SST anomalies during dipole events. J. Mar. Res. 64, 853–880 (2006).

[b22] Grunseich G., Subrahmanyam B., Murty V. S. N. & Giese B. S. Sea surface salinity variability during the Indian Ocean Dipole and ENSO events in the tropical Indian Ocean. J. Geophys. Res. 116, C11013, doi: 10.1029/2011JC007456 (2011).

[b23] Yuhong Z., Yan D., Shaojun Z., Yali Y. & Xuhua C. Impact of Indian Ocean Dipole on the salinity budget in the equatorial Indian Ocean. J. Geophys. Res. 118, 4911–4923, doi: 10.1002/jgrc.20392 (2013).

[b24] Durand F., Alory G., Dussin R. & Reul N. SMOS reveals the signature of Indian Ocean Dipole events. Ocean. Dyn. 63, 1203–1212 (2013).

[b25] Nyadjro E. S. & Subrahmanyam B. SMOS mission reveals the salinity structure of the Indian Ocean Dipole. IEEE,Geosci. Remote. Sens. Lett. 11, 1564–1568 (2014).

[b26] Shi W. Estimation of heat and salt storage variability in the Indian Ocean from TOPEX/Poseidon altimetry. J. Geophys. Res. 108, 3214, doi: 10.1029/2001JC001244 (2003).

[b27] Cooper N. S. The effect of salinity on tropical ocean models. J. Phys. Oceanogr. 18, 697–707 (1988).

[b28] de Boyer Montégut C., Madec G., Fischer A. S., Lazar A. & Iudicone D. Mixed layer depth over the global ocean: An examination of profile data and a profile‐based climatology. J. Geophys. Res. 109, C12003, doi: 10.1029/2004JC002378 (2004).

[b29] Dong C., Idica E. Y. & McWilliams J. C. Circulation and multiple-scale variability in the Southern California Bight. Prog. Oceanogr. 82, 168–190 (2009).

[b30] Shchepetkin A. F. & McWilliams J. C. The regional oceanic modeling system (ROMS): A split-explicit, free-surface, topography-following-coordinate oceanic model. Ocean. Model. 9, 347–404 (2005).

[b31] Rao R. & Sivakumar R. Seasonal variability of sea surface salinity and salt budget of the mixed layer of the north Indian Ocean. J. Geophys. Res. 108, 3009, doi: 10.1029/2001JC000907 (2003).

[b32] Da-Allada C. Y., Gaillard F. & Kolodziejczyk N. Mixed-layer salinity budget in the tropical Indian Ocean: Seasonal cycle based only on observations. Ocean. Dyn. 65, 845–857 (2015).

