# Supplementary material for: A new dipole index of the salinity anomalies of the tropical Indian Ocean
Source: Sci Rep. 2016 Apr 7;6:24260. doi: 10.1038/srep24260 (PMC4823653; doi:10.1038/srep24260)
Supplement: Supplementary Information [file srep24260-s1.pdf]

Supplementary Information for

A new dipole index of the salinity anomalies of the tropical Indian Ocean

Junde Li[1,2], Chujin Liang[1], Youmin Tang[3, 1*], Changming Dong[4,5], Dake Chen[1], Xiaohui Liu[1], Weifang Jin[1]

[1]State Key Lab of Satellite Ocean Environment Dynamics, Second Institute of Oceanography, State Oceanic Administration, Hangzhou, China

[2]College of Physical and Environmental Oceanography, Ocean University of China, Qingdao, China

[3]Environmental Science and Engineering, University of Northern British Columbia, Prince George, British Columbia, Canada

[4]Oceanic Modeling and Observation Laboratory, Nanjing University of Information Science and Technology, Nanjing, China

[5]Department of Atmospheric and Oceanic Sciences, University of California, Los Angeles, California, USA

***** Corresponding author: Dr. Youmin Tang, 3333 University Way, Prince George, British Columbia, Canada, V2N 4Z9, Email: ytang@unbc.ca

Supplementary figures

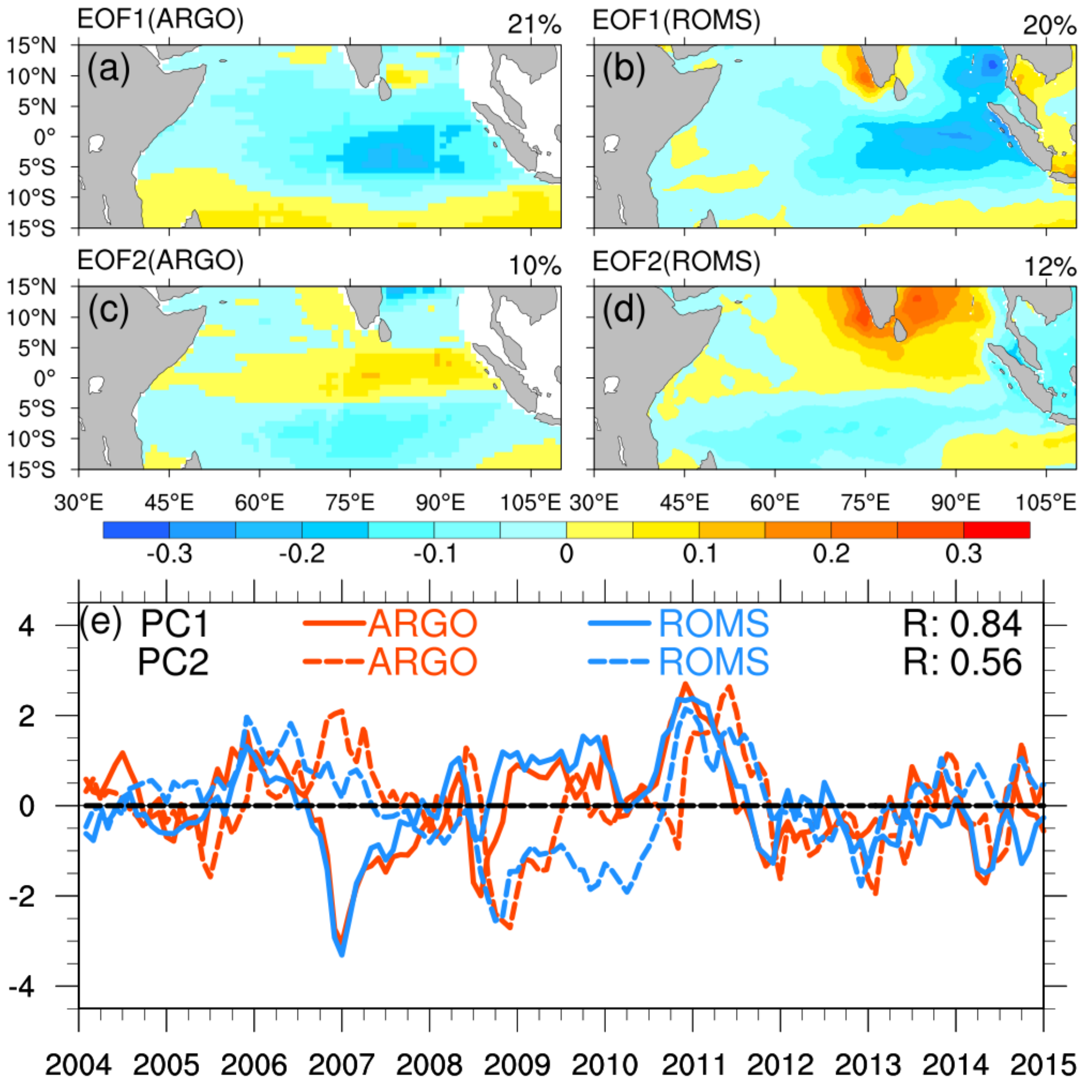

Figure S1| Spatial patterns of the first and second EOF mode and their corresponding principal components (PCs) for the monthly SSS anomalies from 2004 to 2014. (a) and (b) are the first EOF patterns obtained from ARGO and ROMS, respectively. (c) and (d) are the second EOF patterns obtained from ARGO and ROMS, respectively. The maps were generated in The NCAR Command Language (Version 6.3.0) [Software]. (2016). Boulder, Colorado: UCAR/NCAR/CISL/TDD. http://dx.doi.org/10.5065/D6WD3XH5. (e) Corresponding PCs of the first (solid) and the second (dash) EOF mode from ARGO (red line) and ROMS (blue line). R is the correlation coefficient between the both PCs.

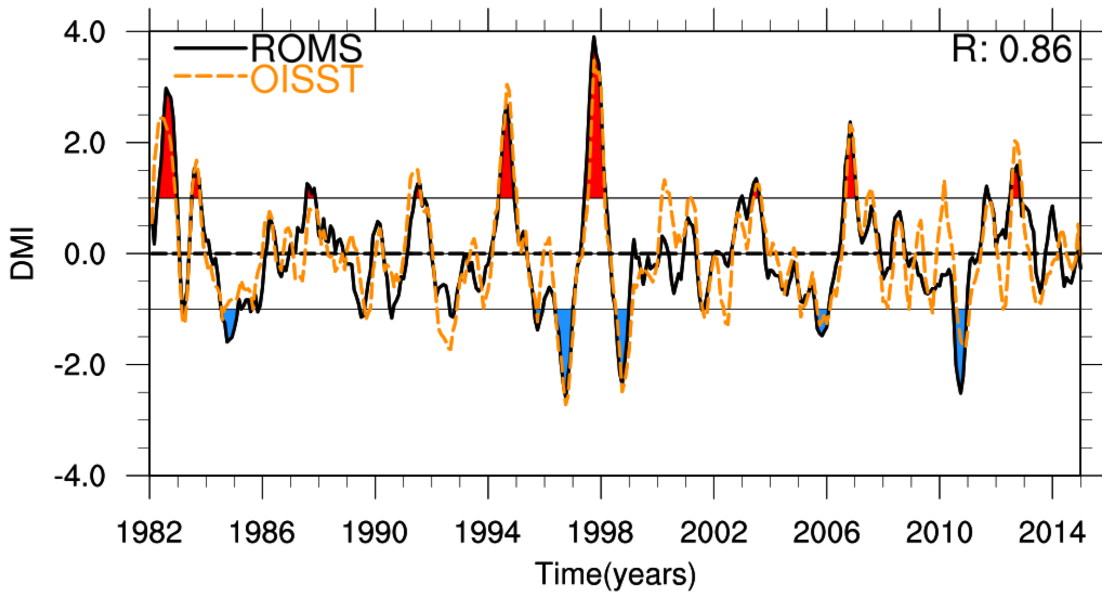

Figure S2| Dipole mode index (DMI) obtained from ROMS (black solid line) and OISST (orange dashed line) SST from 1982 to 2014. The DMI time series have been normalized by their standard deviations (std); the values greater than 1.0 std of ROMS SST are highlighted in red, and the values less than 1.0 std are highlighted in blue. The DMI time series are filtered by a bandpass filter of 5-84 months. All plots were generated in The NCAR Command Language (Version 6.3.0) [Software]. (2016). Boulder, Colorado: UCAR/NCAR/CISL/TDD. http://dx.doi.org/10.5065/D6WD3XH5.

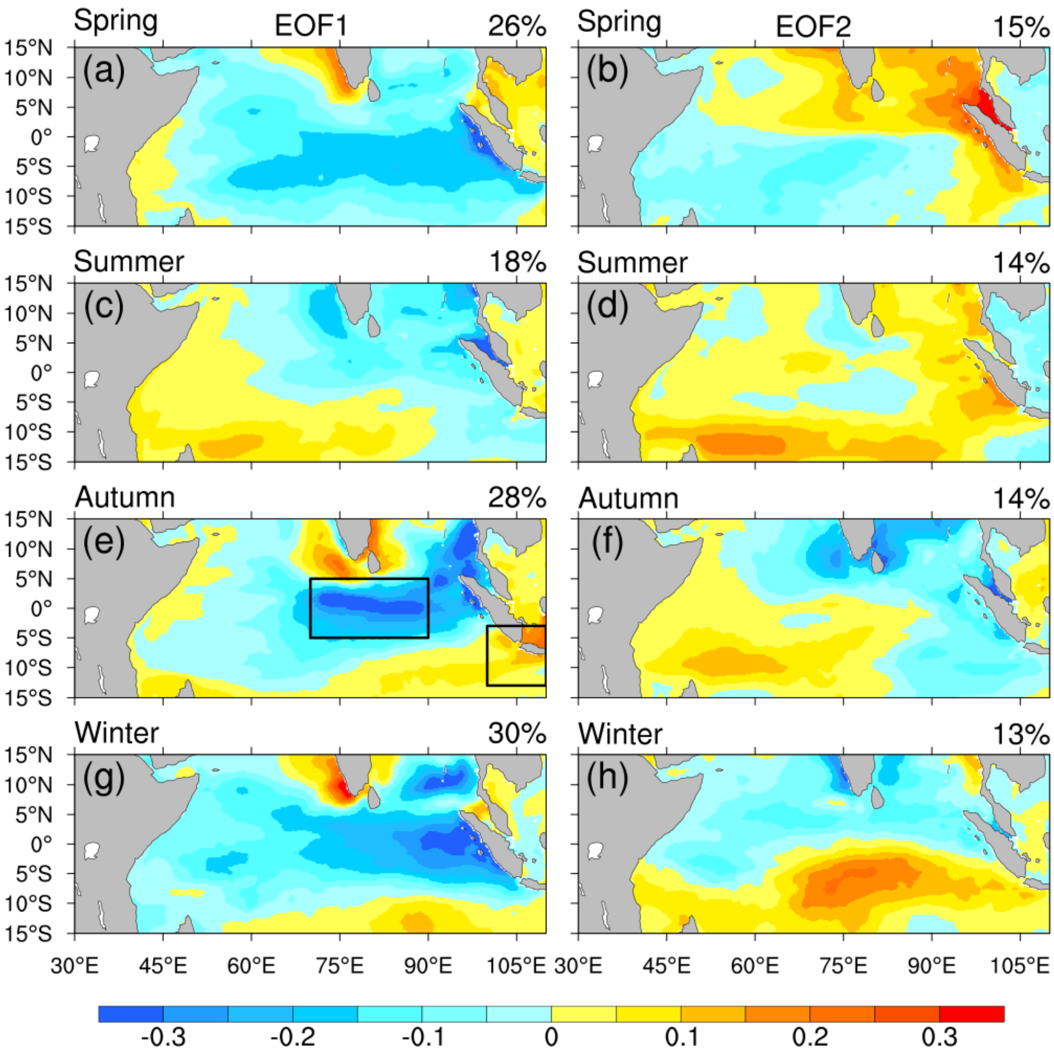

Figure S3| Spatial patterns of the first and second EOF mode for SSS anomalies obtained using ROMS seasonal data from 1982 to 2014 for spring (March-May) (a & b), summer (June-August) (c & d), autumn (September-November) (e & f), and winter (December-February) (g & h). The black boxes in (e) show the dipole locations: the central equatorial Indian Ocean (CEIO: 70°E-90°E, 5°S-5°N) and the Sumatra-Java coast (SJC: 100°E-110°E, 13°S-3°S). The maps were generated in The NCAR Command Language (Version 6.3.0) [Software]. (2016). Boulder, Colorado: UCAR/NCAR/CISL/TDD. http://dx.doi.org/10.5065/D6WD3XH5.

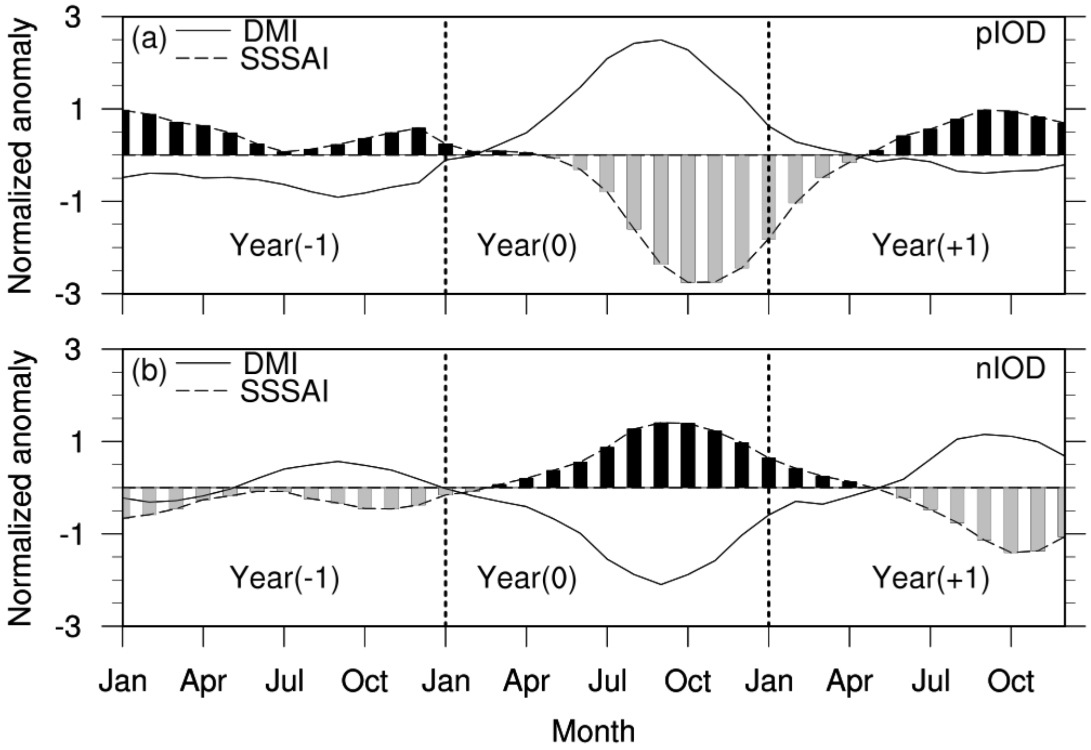

Figure S4| Temporal evolution of DMI (solid line) and SSSAI (dashed line) time series for composited IOD events. (a) Evolution of DMI and SSSAI for composited positive IOD events (1982, 1994, 1997, 2006 and 2012) from the previous IOD year (-1), IOD year (0) to the following IOD year (+1). (b) Same as (a), but for negative IOD events (1984, 1996, 1998, 2005 and 2010). SSSAI are indicated with black bars (>0) and grey bars (<0) in both (a) and (b). All plots were generated in The NCAR Command Language (Version 6.3.0) [Software]. (2016). Boulder, Colorado: UCAR/NCAR/CISL/TDD. http://dx.doi.org/10.5065/D6WD3XH5.

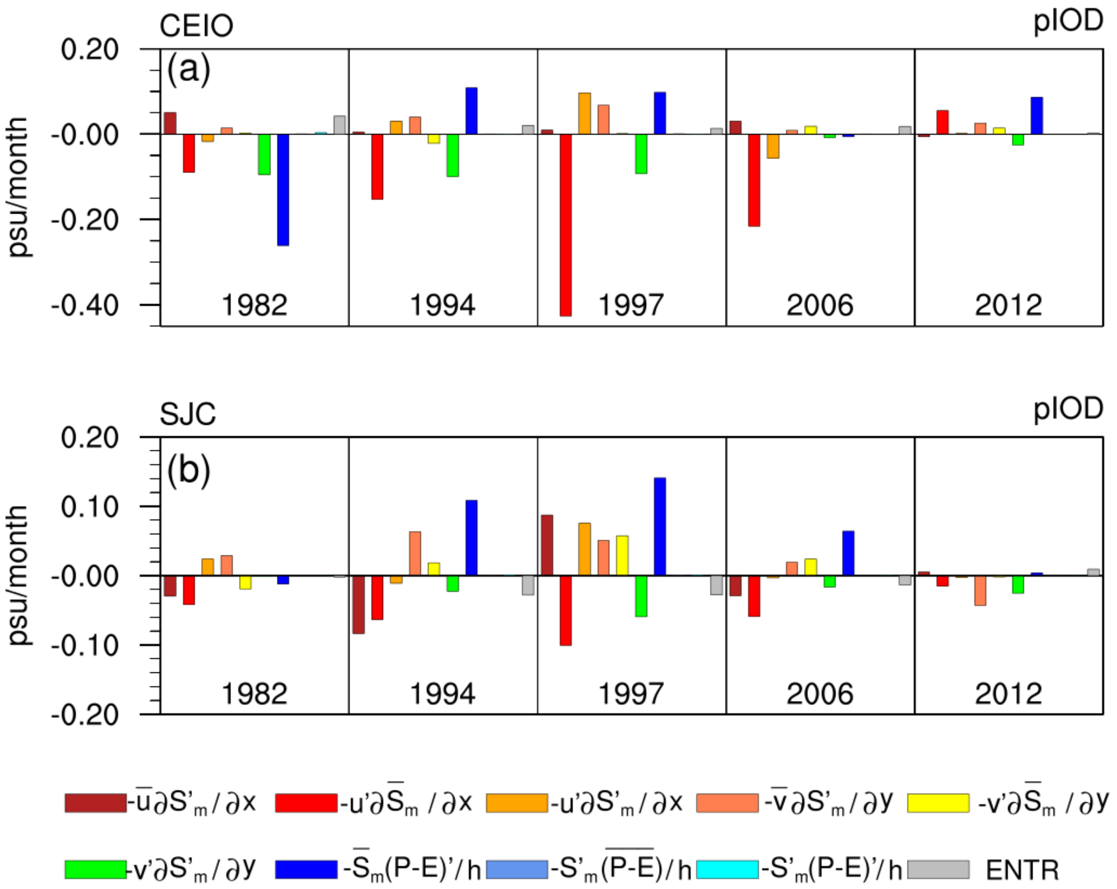

Figure S5| Salinity budget components averaged over August-September. (a) Averaged within the CEIO region for each positive IOD event (1982, 1994, 1997, 2006 and 2012). (b) Same as (a), but for SJC. All plots were generated in The NCAR Command Language (Version 6.3.0) [Software]. (2016). Boulder, Colorado: UCAR/NCAR/CISL/TDD. http://dx.doi.org/10.5065/D6WD3XH5.

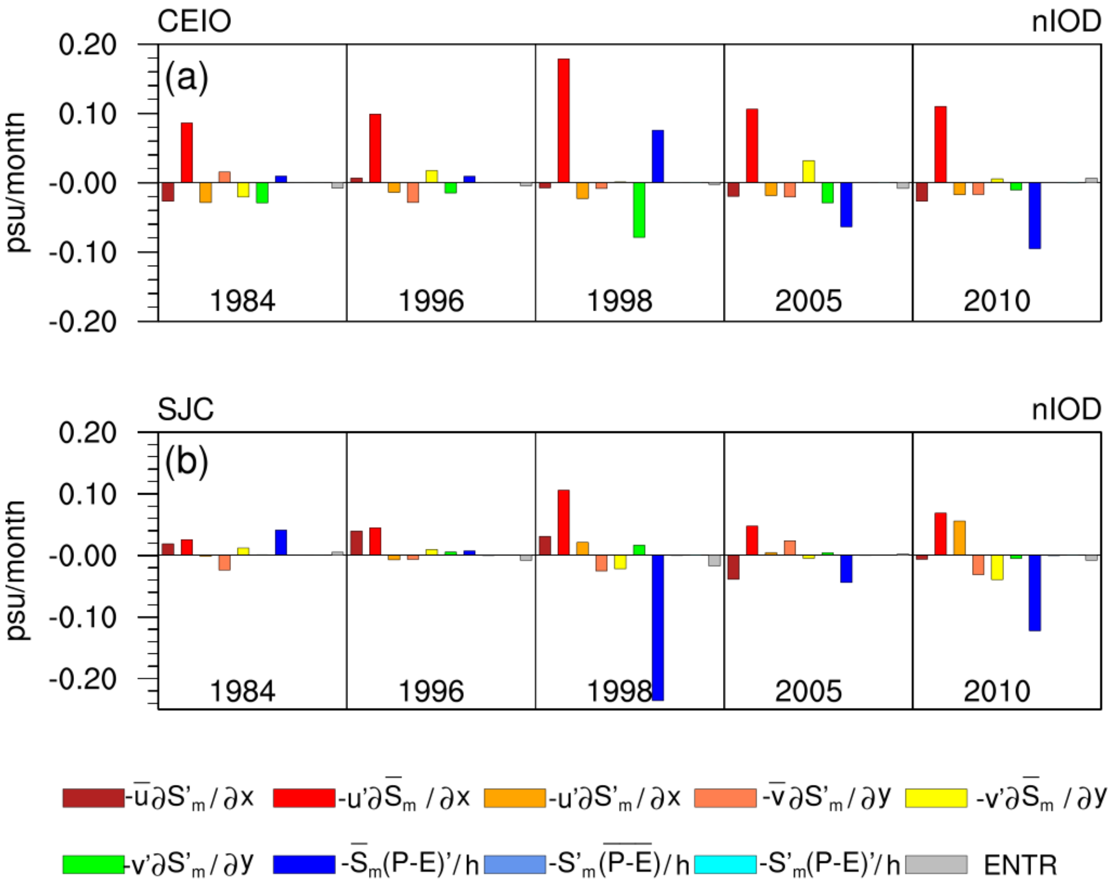

Figure S6| Same as Supplementary Fig. S5, but for each negative IOD event (1984, 1996, 1998, 2005 and 2010). All plots were generated in The NCAR Command Language (Version 6.3.0) [Software]. (2016). Boulder, Colorado: UCAR/NCAR/CISL/TDD. http://dx.doi.org/10.5065/D6WD3XH5.
